# Beneficial Effects of Qingzixiaoban Granule on Henoch–Schönlein Purpura Nephritis Mice through Inhibiting Immune Complex Deposition and Th2 Immunodeviation

**DOI:** 10.1155/2019/3050248

**Published:** 2019-10-16

**Authors:** Hui Yang, Jing Guan, Pei Ma, Yannan Fan, Jinye Bai, Shuyi Li, Jiqiao Yuan, Yecheng Jin, Mingbao Lin, Jianmin Zhang, Qi Hou

**Affiliations:** ^1^State Key Laboratory of Bioactive Substances and Functions of Natural Medicines, Institute of Materia Medica, Chinese Academy of Medical Sciences & Peking Union Medical College, Beijing, China; ^2^Capital Institute of Pediatrics, Beijing, China; ^3^Affiliated Sir Run Run Shaw Hospital, Zhejiang University School of Medicine, Hangzhou, China

## Abstract

**Background:**

Henoch–Schönlein purpura nephritis (HSPN) is the principal cause of morbidity and mortality in Henoch–Schönlein purpura (HSP). However, there is no absolute consensus for the best management of severe HSPN till now. Qingzixiaoban Granule (QZXB GR), a traditional Chinese medicine formula, has been applied to treat HSP in clinical in China. However, the therapeutic effects and potential mechanism of QZXB GR on HSPN is still unknown.

**Methods:**

A Gliadin plus Indian Ink-induced HSPN mice model was established. Renal histopathologic changes and the subcutaneous hemorrhage on left legs were assessed. Hematuria and proteinuria were determined using hemocytometer and bicinchoninic acid assay, respectively. The serum circular immune complex and interleukin-6 were quantified by ELISA. Using blood biochemical analyzer, the renal biochemical parameters, including serum total protein, albumin, creatinine, and blood urea nitrogen, were measured. The deposition of immune complex in renal tissues and the lymphocyte subsets in peripheral blood and spleen was investigated by immunohistochemistry and flow cytometry.

**Results:**

QZXB GR treatment significantly ameliorated renal injury in HSPN mice, by attenuating renal histopathological changes, reducing subcutaneous hemorrhage, decreasing proteinuria/hematuria, regulating renal biochemical parameters, and inhibiting the release of serum interleukin-6. Furthermore, QZXB GR treatment significantly decreased the level of serum circular immune complex, decreased immune complex IgA and IgG deposition in renal tissue, and suppressed Th2 immunodeviation.

**Conclusion:**

QZXB GR could prevent renal injury in HSPN mice, and its renoprotective mechanism might be exerted partly through suppressing immune complexes deposition and Th2 immune deviation.

## 1. Introduction

Henoch–Schönlein purpura (HSP) nephritis (HSPN) is one of the major clinical manifestations (renal injury) and primary cause of mortality and morbidity in HSP [1]. Within 4–6 weeks after initial disease onset, approximately 30–50% of children with HSP progress to HSPN [2], which accounts for 1.8–3% of children with chronic kidney disease and may result in chronic renal failure in 11–38% of patients with severe manifestations and pathologic changes in long-term follow-up [3]. The severity of renal injury is the key factor determining the prognosis of HSPN [1]. Therefore, great efforts are in urgent need for renal injury controlling in children with HSPN. However, till now, there is no absolute consensus for the best management of severe HSPN, and the most effective treatment remains controversial [3]. Furthermore, corticosteroids, immunosuppressants, and anticoagulants have potential side effects, such as oncogenesis, myelosuppression, hemorrhagic cystitis, and interstitial pneumonia [4].

As to this, traditional Chinese medicine (TCM) has shown significant efficacy and advantage in clinical [4] and seems to be an important and novel therapeutic candidate for the treatment of HSPN. In recent years, it has been reported that there were additional positive effects in quite a few trials conducted in China by the use of TCM in conjunction with corticosteroids or immunosuppressive drugs [5, 6]. Many TCM can improve immune function and reduce the associated renal damage through regulating immune balance and remitting hypercoagulability of blood [7].

Qingzixiaoban Granule (QZXB GR), a formula comes from clinical experience for treating HSP in children and adolescents in China, consists of *Indigofera tinctoria* L., *Lithospermum erythrorhizon* Siebold & Zucc, *Salvia miltiorrhiza* Bunge, *Moutan officinalis* (L.) Lindl. & Paxton, *Clematis chinensis* Osbeck, *Agrimonia pilosa* Ledeb., *Kochia scoparia* (L.) Schrad., and *Dictamnus dasycarpus* Turcz. Based on the traditional Chinese medicine theory, the beneficial effects of QZXB GR are related to promote blood circulation and remove blood stasis [8]. However, there are limited data regarding therapeutic effects of QZXB GR on HSPN, even lack of potential mechanism data.

Sometimes, HSPN is referred as immunoglobulin (Ig)A vasculitis or anaphylactoid purpura nephropathy, which tends to present as acute glomerular inflammatory lesions resulted from the glomerular deposition of an abnormally glycosylated IgA1, leading to mesangial proliferative changes [9]. Polyclonal B cells are activated with an increase in IgA-containing complexes that deposit in glomerular mesentery, resulting in mesangial hypercellularity inflammatory cytokine release and extracellular matrix expansion [10] and/or deposit in the small vessels to affect complement activation, increase permeability of vessel wall, and aggravate vascular inflammation [11]; these deposits finally lead to glomeruli and tubules damage [12]. Additionally, the deposition of IgG in mesentery may also be one of the important risk factors in the pathogenesis of renal lesions in HSPN [13]. Therefore, the duration of production, amount, and localization of IgA/IgG circulating immune complexes may be the possible mechanisms of HSPN and responsible for the different presentation and symptoms in clinical.

In addition, cellular immune function disorder, especially helper T (Th) cell subsets disorder, plays a crucial role in HSPN [14]. Th1/Th2 imbalance is an important factor in immune response, Th cells differentiate into Th1 cells to trigger cell-mediated immunity responses and into Th2 cells to trigger the immunity and initiate allergic reactions, respectively [14]. An excessive Th2-dominated response has been characterized in children with HSP [15], which aggravates inflammatory response and promotes cytokines release, stimulating B cell to synthetize and secrete immunoglobulin [16]. The increase in Th2 immunodeviation in peripheral blood causes the development of vessel vasculitis and results in renal microvascular injury in patients with HSPN [14]. Consequently, Th1/Th2 imbalance may be another factor involved in the underlying pathogenesis mechanism of HSPN.

Accordingly, in this study, the therapeutic effects of QZXB GR on HSPN were evaluated; moreover, its potential mechanisms involved in the mitigation of IgA and IgG deposition and the regulation of Th1/Th2 immune imbalance were also investigated.

## 2. Materials and Methods

### 2.1. Drug Preparation

The eight ingredients of QZXB GR (151101, Beijing Shouer Pharmaceutical Factory) were *Indigofera tinctoria* L. (dried leaves and stems; 125 g), *Lithospermum erythrorhizon* Siebold & Zucc. (dried roots; 375 g), *Salvia miltiorrhiza* Bunge (dried rhizome; 500 g), *Moutan officinalis* (L.) Lindl. & Paxton (dried root bark; 500 g), *Clematis chinensis* Osbeck (dried rhizome; 375 g), *Agrimonia pilosa* Ledeb. (dried aerial parts; 500 g), *Kochia scoparia* (L.) Schrad. (dried ripe fruit; 250 g), and *Dictamnus dasycarpus* Turcz. (dried root bark; 250 g). Briefly, *Moutan officinalis* (L.) Lindl. & Paxton were distilled in water (1 : 16, w/v) to extract paeonol, followed by the collection of the resulting distilled Liquid 1 and Herb Residues 1. Inclusion complex of paeonol with *β*-cyclodextrin was prepared. *Indigofera tinctoria* L., *Lithospermum erythrorhizon* Siebold & Zucc., and *Salvia miltiorrhiza* Bunge were dipped (40°C) extracting 2 times in 95% ethanol (1 : 10 and 1 : 8, w/v) for 24 hours, respectively, filtered, and concentrated (liquid : medicinal material ratio of 1 : 1.00–1.05), followed by the collection of the resulting Extraction 1 and Herb Residues 2. *Clematis chinensis* Osbeck, *Agrimonia pilosa* Ledeb., *Kochia scoparia* (L.) Schrad., and *Dictamnus dasycarpus* Turcz. were added to the Herb Residues 1 and 2. They were cooked twice with 11 and 9 times the volume of water for 30 min, respectively, filtered, combined with Liquid 1, and concentrated (liquid : medicinal material ratio of 1 : 1.20–1.25) to obtain Extraction 2. Extraction 2, mannitol, dextrin, aspartame, and *β*-cyclodextrin inclusion complex of paeonol were mixed at a ratio of 1 : 0.2 : 0.4 : 0.005 : 1 to prepare granules, dried, and sprayed with Extraction 1 below 60°C. Finally, the mixture was sprayed 0.1% orange flavor and mixed to produce the QZXB GR formula.

The following drugs were used: Xuening capsule (XN Caps, 150803, Yantai Zhongzhou Pharmacy, China) and mycophenolate mofetil (MMF, SH0065, Shanghai Roche Pharmaceuticals, China).

### 2.2. Animals

Male and female KM mice (*n* = 64 per gender, 14–16 g, 3-4 weeks) were obtained from Vital River Experimental Animal Services (Beijing, China) and housed under pathogen-free conditions with controlled temperature of 24 ± 2°C and humidity of 60 ± 5% with a 12 h light/dark cycle. Because HSP most commonly occurs in children, young mice (14–16 g) were used in this study, and similar results were obtained using mice of different genders. Standard laboratory chow and water were provided *ad libitum*. All animal experiments were approved by the Experimental Animal Care and Use Committee of the Institute of Materia Medica, Chinese Academy of Medical Sciences & Peking Union Medical College (No. 20160612).

### 2.3. Gliadin plus Indian Ink-Induced HSPN in Mice and Treatment

**Figure 1:**
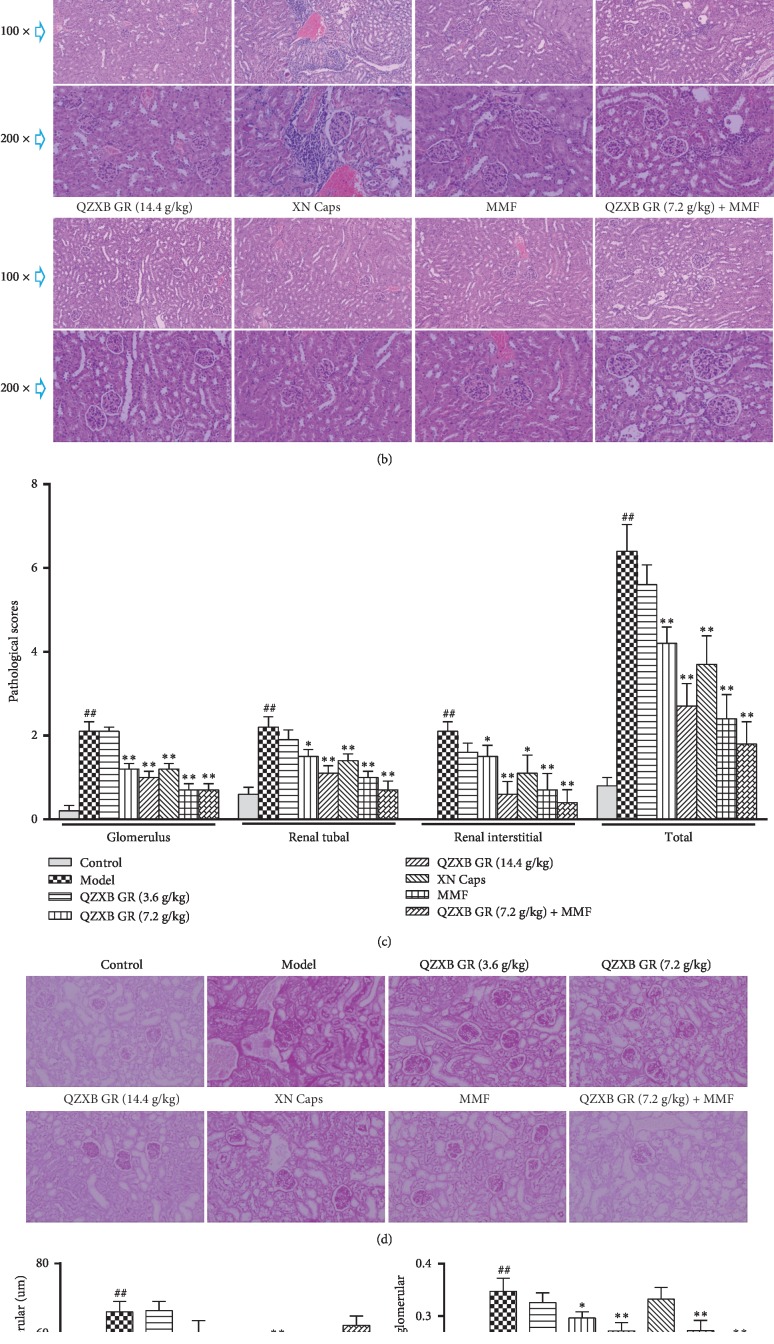
QZXB GR attenuated renal pathological damages in HSPN mice. (a) Timeline for the development and treatment process of HSPN. (b) Images of pathological changes in renal tissues stained by H&E staining (magnified ×100 and ×200). (c) Quantification of pathological scoring of renal injury in H&E staining. (d) Images of pathological changes in renal tissues with PAS staining (magnified ×200). (e) Quantification of glomerular diameters in PAS-stained renal sections. (f) Quantification of optical density in PAS-stained renal sections. Data were presented as means ± SEM, *n* = 10. ^#^*P* < 0.05 and ^##^*P* < 0.01*versus* control group, ^*∗*^*P* < 0.05 and ^*∗∗*^*P* < 0.01*versus* HSPN model group.

Mice were randomly divided into 8 groups: control group, model group, QZXB GR (3.6 g/kg/day, 6 times of dose in clinical practice) group, QZXB GR (7.2 g/kg/day) group, QZXB GR (14.4 g/kg/day) group, XN Caps (0.84 g/kg/day, 12 times of dose in clinical practice) group, MMF (0.3 g/kg/day, 12 times of dose in clinical practice) group, and QZXB GR (7.2 g/kg/day) + MMF (0.3 g/kg/day) group. All groups except control received tail-vein injection of Indian Ink (Solarbio, China) 0.5 mg/10 g once a week for 3 weeks, followed by intragastric administration of 0.5 ml of 1 mg/ml Gliadin (Sigma-Aldrich, USA) in 6 mmol/l HCl every two days for 11 weeks, and finally, tail-vein injection of the mixture of 0.1 ml PBS and 0.1 ml of freshly prepared 10 mg/ml Gliadin every day for three days. Control group received vehicle. The detail of experimental design is shown in Figure 1(a).

All groups except control and model administrated intragastrically corresponding drugs at different doses every day during 9–16 week. Control and model groups received equal amount of saline. At the end of 16^th^ week, mice were sacrificed via euthanasia. Skin, urine, blood, and kidney samples were collected for further analysis.

### 2.4. Histopathologic Assessment of Kidney Tissues

Kidneys were immersion-fixed in 4% paraformaldehyde, dehydrated, paraffin embedded, and sectioned at 4 *μ*m for hematoxylin and eosin (H&E) staining and periodic acid-Schiff base (PAS) staining. The glomerular damage and renal tubular injury were evaluated, respectively, in H&E stain as follows: (1) glomerular damage, such as glomerular interstitial (mesangial) proliferation, renal capsule cavity shrinkage or disappearance, glomus atrophy, and basement membrane thickening; (2) renal tubular injury, such as vacuolar degeneration of renal tubular epithelial cells, tubular atrophy, and cast. A pathological score was given according to the injury ratio of glomerulus or renal tubule: 0 = no injury; 1 = 1%–25% injury ratio; 2 = 26%–50% injury ratio; and 3 = 51%–100% injury ratio. The renal interstitial inflammation in H&E stain was scored as follows: 0 = normal, 1 = slight, 2 = moderate, and 3 = severe. Besides, mean diameter and optical density of glomerular were digitally quantified in PAS stain.

### 2.5. Subcutaneous Hemorrhage Evaluation

The skin of left hindlimb (about 1.5 cm × 1.5 cm) was obtained and pictured for subcutaneous hemorrhage score according to the following criteria: 0 = no subcutaneous capillaries, petechiae, or ecchymosis; 1 = visible capillaries but no obvious telangiectasia; 2 = two or more sites with obvious telangiectasia; and 3 = visible subcutaneous hemorrhage and ecchymosis.

### 2.6. Urine Sample Collection and Analysis

Fresh urine microscopy for RBC, WBC, and CAST was conducted every two weeks during weeks 9–16. The 24-hour urine was collected using metabolic cages before scarification to examine the volume of urine and 24 h urinary protein excretion with BCA protein assay kit (Solarbio, China).

### 2.7. Serum Sample Collection and Analysis

Blood samples were collected from the eyeball of mice, clotted, and centrifuged at 1,000 *g* for 20 min at 20°C. Levels of interleukin-6 (IL-6) and circular immune complex (CIC) were assessed by mouse IL-6 enzyme-linked immunosorbent assay (ELISA) kit (Biolegend, USA) and mouse CIC ELISA kit (Senbeijia Biological Technology, China), respectively. Total protein (TP), albumin (ALB), creatinine (Cre), and blood urea nitrogen (BUN) in serum were evaluated by blood biochemical analyzer (TOSHIBA, Japan) with commercial kits (Biosino Biotechnology and Science, China) according to the manufacturer's protocols.

### 2.8. Immunohistochemistry Studies of IgA and IgG in Kidney Tissues

The paraffin-embedded kidney sections (5 *μ*m) were rehydrated, antigens retrieved using heated citrate, incubated with rabbit antimouse IgA and IgG (Bethyl Laboratories, USA), and visualized using horseradish peroxidase- (HRP-) coupled secondary antibodies (Cell Signaling Technology, USA). The positive staining of mesangial, vascular loop, tubular basement membrane (TBM), and arteriole wall was evaluated by integrated optical density, and a score was given as follows: 0 = no positive staining cells; 1 = 1%–25% positive staining; 2 = 26%–50% positive staining; and 3 = 51%–100% positive staining.

### 2.9. Flow Cytometrical Analysis

A single-cell suspension was prepared from blood sample or spleen sample. Blood cells were incubated with anti-CD3-PE and anti-CD19-PE-Cy7 monoclonal antibody (BD Biosciences, USA) at 4°C for 30 min. Spleen cells were incubated with anti-CD3-PE, anti-CD4-FITC, and anti-CD8-Percp-Cy5.5 monoclonal antibody (BD Biosciences, USA) at 4°C for 30 min. For intracellular staining, cells were fixed and permeabilized after surface staining, and anti-IL-4-APC and anti-IFN-γ-PE-Cy7 (eBioscience, USA) were then added to stain Th2 cells and Th1 cells. Stained cells were analyzed by flow cytometric analysis using a flow cytometer (ACEA NovoCyte 2060R, China).

### 2.10. Statistical Analysis

Data were expressed as mean± standard error (SEM). As the normality test by Kolmogorov–Smirnov test (K-S test) was passed, data were analyzed using Student's *t*-test for comparison between two groups and one-way ANOVA for multiple groups followed by Fisher's least significant difference (LSD) test, otherwise, using Mann–Whitney *U* test. Values of *P* < 0.05 were regarded as significant. The above analyses were conducted with GraphPad Prim 6.0 and SPSS 19.0 statistical software.

## 3. Results

### 3.1. QZXB GR Attenuated Renal Pathological Damages in HSPN Mice

Hematoxylin-eosin (H&E) and periodic acid-Schiff (PAS) staining were used to assess the renal histopathologic changes in HSPN mice. A pathological score was used to quantify the glomerular injury, renal tubal injury, and renal interstitial inflammation in H&E staining. As shown in Figures 1(b) and 1(c), QZXB GR (7.2 g/kg and 14.4 g/kg) treatment significantly reduced the pathological scores of glomerulus, renal tubal, and renal interstitial injury compared with the model group (*P* < 0.05 or 0.01), as same as positive control XN Caps and MMF treatment. Furthermore, QZXB GR (7.2 g/kg) combined with MMF treatment behaved more effective in renal tubal, renal interstitial injury, and total pathological scores compared with QZXB GR and MMF single treatment.

PAS staining was used to observe the pathological changes of glomerular basement membrane. As shown in Figures 1(d) and 1(e), compared with the model group, QZXB GR (14.4 g/kg), XN Caps, and MMF treatment significantly decreased HSPN mice glomerular diameter (*P* < 0.01). Additionally, QZXB GR (7.2 and14.4 g/kg) treatment also notably reduced glomerular mean optical density in HSPN mice (*P* < 0.05 or 0.01), as well as that of MMF and QZXB GR (7.2 g/kg) + MMF treatment (*P* < 0.01). These results showed potential protective effects of QZXB GR on renal tissue pathological damages in HSPN mice.

### 3.2. QZXB GR Reduced Subcutaneous Hemorrhage, Proteinuria, and Hematuria in HSPN Mice

**Figure 2:**
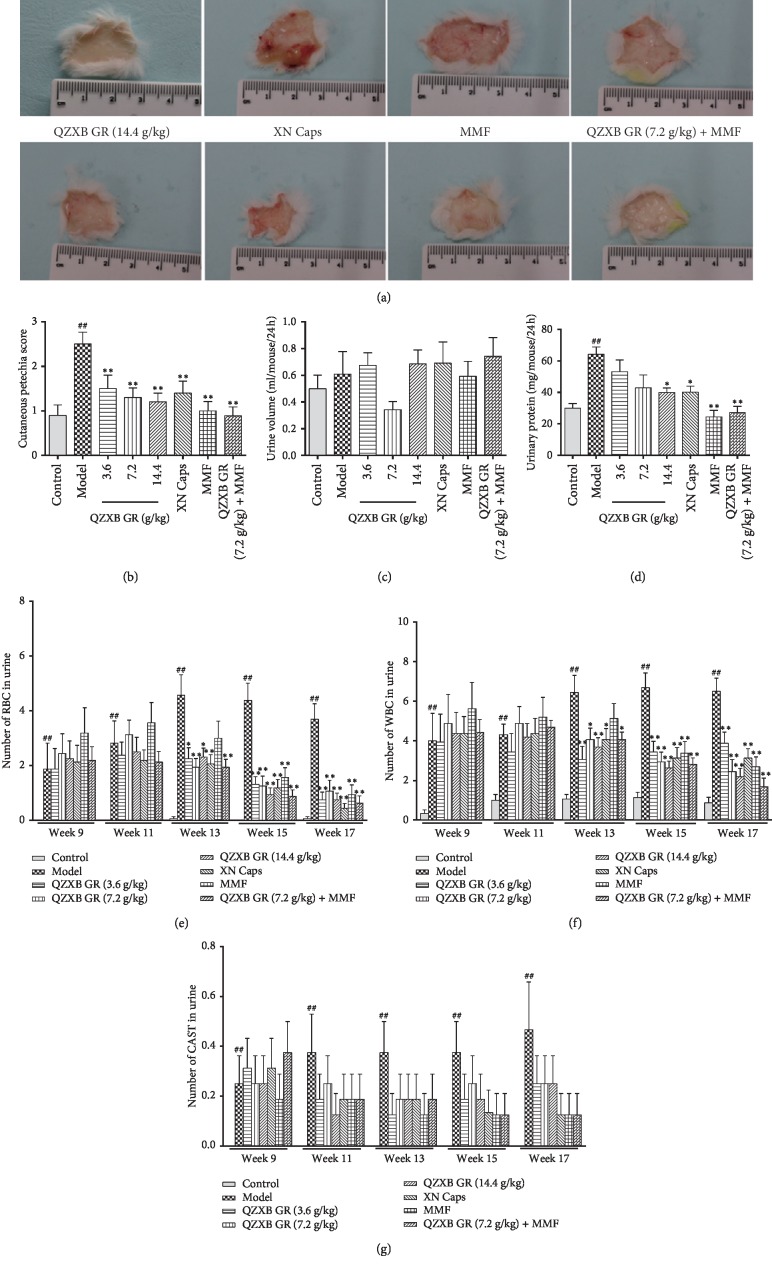
QZXB GR reduced subcutaneous hemorrhage, hematuria, and proteinuria in HSPN mice. (a) Images of subcutaneous hemorrhage in HSPN mice (*n* = 10). (b) Quantitative scoring of subcutaneous hemorrhage in HSPN mice (*n* = 10). (c) 24-hour urine volume after the last treatment (*n* = 8). (d) 24-hour urinary protein excretion after the last treatment (*n* = 8). (e) Numbers of RBC in fresh urine of HSPN mice from 9^th^ week (*n* = 16). (f) Numbers of WBC in fresh urine of HSPN mice from 9^th^ week (*n* = 16). (g) Numbers of CAST in fresh urine of HSPN mice from 9^th^ week (*n* = 16). Data were presented as means ± SEM. ^#^*P* < 0.05 and ^##^*P* < 0.01*versus* control group; ^*∗*^*P* < 0.05 and ^*∗∗*^*P* < 0.01*versus* HSPN model group.

As shown in Figures 2(a) and 2(b), the subcutaneous hemorrhage on left legs was pronounced in HSPN model mice, which might be significantly decreased with QZXB GR treatment (*P* < 0.01), as well as XN Caps and MMF treatment. Furthermore, the subcutaneous hemorrhage was collaboratively inhibited by combination treatment of QZXB GR (7.2 g/kg) and MMF.

Then, 24-hour urine was collected, and its volume and protein excretion were determined. As shown in Figure 2(c), there were no significant changes in urine volume among all experimental groups (*P* > 0.05). However, compared with the control mice, 24 h urinary protein levels were significantly increased in HSPN model mice (*P* < 0.01), which might be significantly reduced with QZXB GR (14.4 g/kg) treatment (*P* < 0.05), as well as XN Caps, MMF, and QZXB GR (7.2 g/kg) + MMF treatment (*P* < 0.05 or 0.01; Figure 2(d)).

From 9^th^ week, the numbers of RBC (Figure 2(e)), WBC (Figure 2(f)), and CAST (Figure 2(g)) in fresh urine were counted by microscopy every two weeks. Compared with the control group, the counts of urinary RBC, WBC, and CAST were significantly increased in the HSPN model group (*P* < 0.01). QZXB GR treatment might significantly decrease urinary RBC and WBC count after 13^th^ week (*P* < 0.05 or 0.01), as well as XN Caps treatment, whereas MMF treatment exerted the effect after 14^th^ week. The effects were facilitated by combinatory use of QZXB GR with MMF.

### 3.3. QZXB GR Decreased the Levels of Serum CIC, IL-6, and Regulated Renal Biochemical Parameters in HSPN Mice

**Figure 3:**
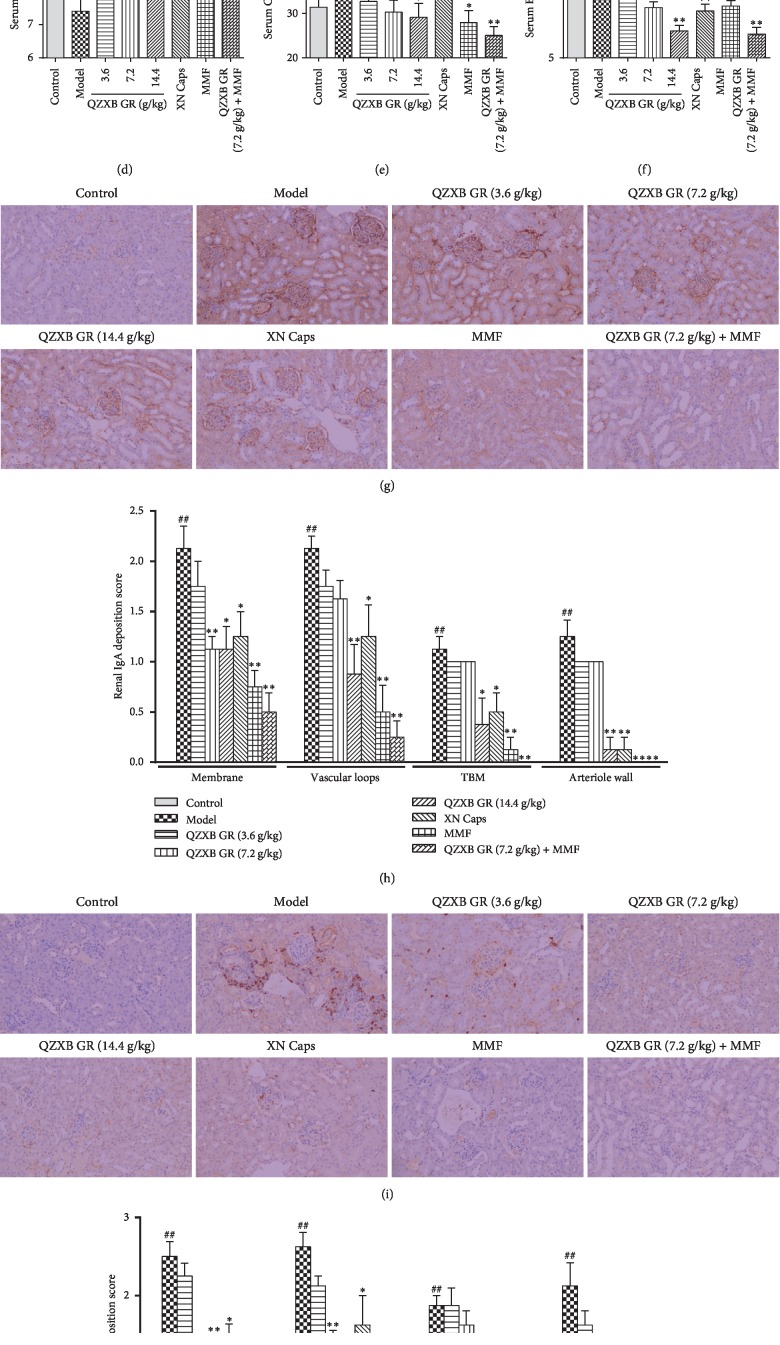
QZXB GR decreased the levels of CIC, IL-6, renal biochemical parameters in the serum and suppressed immune complex deposition in HSPN mice kidneys. (a) The level of serum CIC in HSPN mice. (b) The level of serum IL-6 in HSPN mice. (c) The level of serum TP in HSPN mice. (d) The level of serum ALB in HSPN mice. (e) The level of serum Cre in HSPN mice. (f) The level of serum BUN in HSPN mice. (g) Representative photomicrographs of IgA deposition (×200). (h) Quantitative scoring of IgA deposition. (i) Representative photomicrographs of IgG deposition (×200). (j) Quantitative scoring of IgG deposition. Data were presented as means ± SEM, *n* = 8. ^#^*P* < 0.05 and ^##^*P* < 0.01*versus* control group, ^*∗*^*P* < 0.05 and ^*∗∗*^*P* < 0.01*versus* HSPN model group.

The serum CIC and IL-6 were determined by ELISA. As shown in Figure 3(a), compared with the model group, QZXB GR (14.4 g/kg) treatment significantly decreased the serum CIC (*P* < 0.05), similar to that of MMF, and a superimposed effect might be observed in combination treated group. As shown in Figure 3(b), QZXB GR treatment could also significantly inhibit the levels of serum IL-6 in HSPN mice (*P* < 0.01), as well as XN Caps, MMF, and QZXB GR (7.2 g/kg) + MMF treatment.

Furthermore, the renal biochemical parameters, including serum TP (Figure 3(c)), ALB (Figure 3(d)), Cre (Figure 3(e)), and BUN (Figure 3(f)), were measured by blood biochemical analyzer. Compared with the control mice, the serum TP and ALB were significantly decreased, whereas serum BUN was markedly increased in HSPN mice. The serum Cre was also increased in HSPN mice but without significance compared with the control mice. Compared with the HSPN model group, QZXB GR treatment might significantly improve the renal function in HSPN mice, by elevating the serum TP and ALB, decreasing the serum Cre and BUN.

### 3.4. QZXB GR Suppressed the Immune Complexes IgA and IgG Deposition in HSPN Mice Kidneys

Immunohistochemistry analysis was used to investigate the effects of QZXB GR on the deposition of immune complex in renal tissues of HSPN mice. As shown in Figure 3, compared with the control mice, large amounts of IgA (Figures 3(g) and 3(h)) and IgG (Figures 3(i) and 3(j)) deposition could be observed in the mesentery, vascular loop, tubular basement membrane (TBM), and arteriole wall in HSPN model mice (*P* < 0.01). While in contrast to which, QZXB GR treatment might significantly inhibit IgA and IgG deposition (*P* < 0.05 or <0.01), as well as XN Caps and MMF treatment. Moreover, combination treatment showed a superimposed effect in renal IgA and IgG deposition in HSPN mice.

### 3.5. QZXB GR Inhibited the Th2 Immunodeviation in HSPN Mice

**Figure 4:**
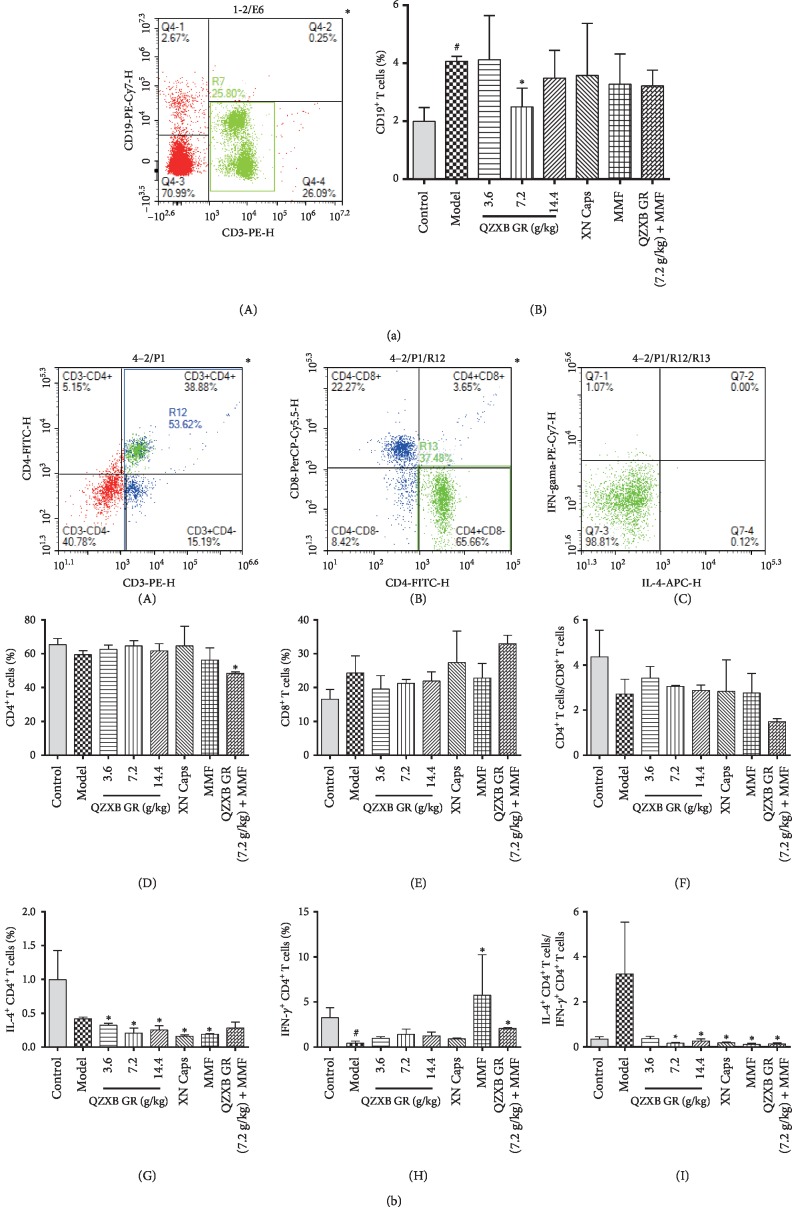
Effects of QZXB GR on lymphocyte subsets in HSPN mice. (a) The lymphocyte subsets in the peripheral blood. (A) Lymphocytes were stained with anti-CD3 and anti-CD19. (B) Quantification of CD19+ B cells. (b) The lymphocyte subsets in the spleen lymphocytes. (A–C) The gating strategy. (D) The percentage of CD4+ T cells. (E) The percentage of CD8+ T cells. (F) The ratio of CD4+ T cells/CD8+ T cells. (G) The percentage of CD4+ IL-4+ T cells. (H) The percentage of CD4+ IFN-*γ*+ T cells, and (I) the ratio of CD4+ IL-4+ T cells/CD4+ IFN-*γ*+ T cells were shown. Data were presented as means ± SEM, *n* = 3. ^#^*P* < 0.05 and ^##^*P* < 0.01 versus control group; ^*∗*^*P* < 0.05 and ^*∗∗*^*P* < 0.01 versus HSPN model group.

Flow cytometric analysis was used to analyze the lymphocyte subsets in peripheral blood and spleen in HSPN mice. As shown in Figure 4(a), the proportion of CD19+ B cells was markedly increased in peripheral blood of HSPN model mice (*P* < 0.05), while QZXB GR (7.2 g/kg) treatment might significantly inhibit the increase (*P* < 0.05). However, no significance was observed in the proportion of IL4^+^ T cells and IFN-*γ*^+^ T cells in peripheral blood (data not shown).

Although there was no significance, the ratio of IL4^+^ T cells/IFN-*γ*^+^ T cells was markedly elevated in HSPN mice, showing a Th2 immunodeviation in spleen lymphocyte subsets (Figure 4(b)), while QZXB GR treatment might significantly recovere the Th1/Th2 disorder, as well as XN Caps, MMF, and QZXB GR (7.2 g/kg) + MMF treatment.

## 4. Discussion

Nephritis is the principal cause of morbidity and mortality in HSP [17]. Although many therapies have been reported, their limited effectiveness and adverse effects highlight the urgent need for new drugs for HSPN treatment. However, to date, animal model of HSPN was rarely reported, which limits the advance research in the pathogenesis and treatment of HSPN. In this study, a HSPN mice model was established using Gliadin plus Indian Ink, referring to the IgAN model, for the similarity of immunological and pathological characteristics between HSPN and IgAN [13]. The dietary antigen Gliadin was applied to stimulate mucosal immune system, leading to the sustainable production of immunoglobulin in peripheral blood and the deposition of immunoglobulin in mesangial [18]. Indian Ink was used to block reticuloendothelial system (RES) and cause RES clearance dysfunction, which may prevent the clearance of IgA [19, 20]. In this study, many features in the mice model were found to be consistent with characteristics of HSPN patients in clinical. Therefore, the mice model may partially be used as a tool for understanding the potential pathological mechanism and developing therapeutic strategy of HSPN.

Using the HSPN mice model, the effect and potential mechanism of QZXB GR were evaluated in this study. Moreover, XN caps (a TCM which consists of peanut seed coat extract for HSPN treatment in clinical [18, 21]) and MMF (an immunosuppressive agent used to alleviate HSPN renal tissue injury [1]) were used as positive control drugs to ensure the sensitivity of this model. The results in this study suggested that QZXB GR was able to recover the renal injury from HSPN in mice, by alleviating renal pathological injury, inhibiting serum IL-6 and CIC, as well as XN caps and MMF administration. Furthermore, in this study, the results also suggested that combinatory administration of QZXB GR and MMF might exhibit a superimposed effect in HSPN mice, which were consistent with that multiple drug combination therapy was effective in ameliorating proteinuria and pathological severity in clinical setting [1, 22].

In HSPN, the long-term prognosis is largely determined by the extent of renal pathological injury, characterized by mesangial injury, mesangial proliferation, crescent formation, and interstitial inflammation [22]. In this study, H&E staining was used to determine the histopathologic pathological score changes in glomerular injury, renal tubal injury, and renal interstitial inflammation, whereas PAS staining used to observe the changes in glomerular basement membrane. The results showed that QZXB GR treatment significantly alleviated renal pathological injury and mesangial matrix proliferation in HSPN mice.

Petechiae and palpable purpura are the most common skin lesions in HSPN [1]. Purpura is often distributed over the surfaces of the lower legs, arms, and the sides of the buttocks, and subcutaneous bleeding may occur anywhere [23]. In this study, the subcutaneous hemorrhage on left legs in HSPN mice were observed and pictured. Our data showed that subcutaneous hemorrhage in HSPN mice was severe and might be significantly alleviated with QZXB GR treatment.

In this study, microscopic hematuria and renal biochemical parameters were used to determine the renal function changes in HSPN mice. The urinary RBC and WBC are always determined for assessing the HSPN patients' kidney function [24]. The increase in proteinuria is an important symbol of glomerular filtration barrier damage and reflects the severity of renal damage [25]. Serum ALB, BUN, and Cre are classical biomarkers of renal function. Reduced serum ALB and increased serum BUN and Cre concentrations often indicate glomerular injuries, tubule injuries, and glomerular filtration rate decline [26]. The results of this study showed that QZXB GR treatment might significantly reduce the levels of proteinuria, urinary RBC and WBC, increased serum TP and ALB, decreased serum BUN and Cre, implying to reverse the abnormality of these renal biochemical parameters in HSPN mice.

IL-6 as a cytokine induces the maturation of B cells into immunoglobulin-secreting cells and promotes the survival and maintenance of long-lived plasma cells [27]. For serum IL-6 significantly increased in patients, it has been a valuable biomarker for clinical diagnosis of HSP [2]. Data in this study showed that the levels of serum IL-6 were significantly increased in HSPN mice, although QZXB GR treatment significantly prevented the increase, which also seemingly suggested that the beneficial effects of QZXB GR could be associated with its anti-inflammation and modulating B-cell maturation activity.

Furthermore, to extend these findings, the underlying mechanisms on HSPN of QZXB GR were investigated. The clinical features of HSPN suggest that the disease is due to systemic deposition of CIC [28]. IgA is one of the most important risk factors in renal lesions development and progression in HSPN [29], which deposit in glomerular leading to cell proliferation, cytokine release, extracellular matrix production, and renal inflammatory changes [30], and in vessel walls, it may be responsible for symptoms involving the skin (petechiae and palpable purpura), joints, and kidneys [30, 31]. The levels of IgG in children with HSPN are extremely high during purpura development, which deposits in mesangial might be associated with renal injury [13]. The results in this study showed that QZXB GR treatment could significantly decrease the levels of serum CIC and block IgA and IgG deposition in kidney of HSPN mice. These suggested that QZXB GR probably exert its renoprotective effects through reducing the production and deposition of immune complexes in the kidney and decreasing immune complex–mediated injury.

Also, it is generally believed that HSPN patients harbor a disorder in immune balance [32]. B cells are generally considered to take part in regulating the immune response, due to their ability to secrete antibodies (such as IgA, IgG, and IgE) and mediate humoral immune response [24]. In this study, QZXB GR decreased the proportion of CD19+ B cells in peripheral blood and decreased the serum CIC, which was synthetized and secreted by B cells, implying that QZXB GR might reduce the number of B cells and inhibited its synthetic and secretory function. The balance between Th1 and Th2 cells in the progression of HSPN is still controversial. Some reported that a shift to Th1 cells existed in HSPN children [33], whereas others suggested that an excessive activation of Th2 lymphocytes and a decrease in Th1/Th2 ratio existed in children with HSPN [14]. In addition, cytokines secreted by Th2 cells such as IL-4 increased and the ratio of IL-4/IFN-γ was elevated in HSPN patients [16], which may contribute to disease by activating B cells and enhancing immunoglobulin production [34, 35]. The results of this study showed that QZXB GR treatment significantly reserved Th1/Th2 immune balance in HSPN mice, which suggested that QZXB GR probably exert its renoprotective effects partly through reversing Th2-dominated immune responses and maintaining immune balance by reducing the ratio of Th2/Th1.

In conclusion, based on the facts that QZXB GR reduced hematuria/proteinuria, reduced skin purpura, attenuated inflammation, and preserved renal function in HSPN mice by suppressing immune complexes deposition and Th2 immune deviation, we believe that it might be a potential drug candidate for HSPN treatment in clinical. Furthermore, the combination therapy of QZXB GR and MMF brought a light of hope for attenuating HSPN.

## Data Availability

The data used to support the findings of this study are available from the corresponding author upon request.
